# Neurodevelopmental disorders: assessing and training working memory

**DOI:** 10.1186/s40359-025-02912-9

**Published:** 2025-10-21

**Authors:** Paula Ferreira, Carmen David, Cristina Costescu, Lucia Vera, Gerardo Herrera, Sara Lopes, Diana Stilwell, Aristides Ferreira, Diogo Domingues, Joana Brito, Joana Campos, Ana Maria Paiva, Ana Margarida Veiga Simão, Ilona Heldal, Teodor Stefanut, Adrian Rosan, Fátima Trindade

**Affiliations:** 1 Faculty of Psychology (UID/04527: Research Center for Psychological Science), University of Lisbon, Lisbon, Portugal; 2 Special Education Department of Babeș-Bolyai University, Cluj, Romania; 3 IRTIC Institute, University of Valencia, Valencia, Spain; 4 Business Research Unit Iscte, Instituto Universitário de Lisboa, Lisbon, Portugal; 5 Faculty of Psychology, University of Lisbon, Lisbon, Portugal; 6 INESC-ID and Instituto Superior Técnico, University of Lisbon, Lisbon, Portugal; 7 Department of Computer Science, Western Norway University of Applied Sciences (HVL), Bergen, Norway; 8 Computer Science Department, Technical University of Cluj-Napoca, Cluj, Romania; 9 Portuguese Association of Down Syndrome, Lisbon, Portugal; 10 ISPA - Instituto Universitário, APPsyCI - Applied Psychology Research Center Capabilities Inclusion, Lisbon, PT & ISCTE-IUL, Instituto Universitário de Lisboa, Business Research Unit (BRU-IUL), Lisbon, Portugal

**Keywords:** Working memory, Educational platform, Neurodevelopmental disorders, Serious games

## Abstract

It is vital that working memory capacity can be assessed and trained with motivating resources, in a personalized way in children with neurodevelopmental disorders. This paper illustrates the possibility of a serious game, such as WorM, to assess and train children’s working memory capacity. WorM was designed, developed and evaluated through three studies (Study 1: informative, Study 2: validation and Study 3: user studies). The first two qualitative studies employ in-depth interviews and focus group discussions, to identify, argue and validate suitable design aspects guided by interviews from teachers and psychologists. The third study presents the usefulness of WorM based on the Item Response Theory analysis and answers from 23 end-user, typical children. Examples of important design aspects identified in Study 1 revealed the role of captivating interests, customization, short duration, rewards, feedback and clear instructions for designing tasks with right images and sound effects. From these, Study 2 enhanced the role of interactive visual feedback for correct and incorrect sorting, using images, keyboard keys, multiple colors, or the phase of the trial. Study 3 illustrates how Correct Pepper Classification tasks show some variability in difficulty, where the range of difficulties was higher in the game tasks than in the standardized Childhood Executive Functioning Inventory (CHEXI) task, usually applied for identifying the NDD characteristics in the examined users. While both instruments showed good reliability with high Person and Item Separation Reliability and Cronbach’s alpha values, the findings of this paper suggest that serious games can offer promising prospects to provide meaningful contexts to assess and train working memory by the WorM game.

**Supplementary Information:**

The online version contains supplementary material available at 10.1186/s40359-025-02912-9.

## Introduction

Properly designed serious games can provide opportunities for learning effectively and efficiently for children with cognitive impairments. For example, for assessing working memory capacity in children with neurodevelopmental disorders (NDDs) [1–3] since training working memory can improve learning in children with NDDs [4]. In fact, games have the potential to successfully regulate children’s cognitive abilities when used for training and rehabilitation purposes [5]. This study answers a call for the development of appropriate serious games specifically designed for NDD children according to their needs and interests [6]. It also answers a call for more research on determining software efficiency concerning the assessment and training of cognitive skills, such as working memory [5]. Thus, we present WorM, an innovative and calibrated serious game in accordance with children’s needs, with a sustainable theme (eliminating food waste within an eco-farm); and illustrate how this game reliably assesses and trains working memory in children with NDDs.

Much research has predominantly targeted adult populations and neurodegenerative conditions, such as Parkinson’s disease [7, 8]. Consequently, there is a limited body of research specifically addressing neurodevelopmental challenges and the application of serious games to facilitate cognitive development in children [9]. Hence, the main theoretical contributions of the WorM serious game lie in its application within the broader framework of empowering children with NDD’s as independent learners. By integrating serious games into the learning process, this research implies a theoretical foundation based on the idea that motivating resources, such as WorM, can play a crucial role in promoting the development of independent learning skills among children with NDD’s. This aligns with the broader educational theory that emphasizes the importance of engaging and motivating learners to foster their autonomy and ability to identify and meet their own learning needs [10, 11].

## Working memory and children with NDDs

Working memory has been defined as the capacity to direct attentional focusing and processing towards significant goals while avoiding distraction [12, 13]. Moreover, it has been considered as individuals’ capacity to sustain information and process additional data [14]. Working memory has been viewed as the system or mechanism underlying the maintenance of task-relevant information during the performance of a cognitive task [15–17]. In the current study we adopt the Miyake et al. conceptual framework of working memory systems [12, 17] that identifies three executive functional components of working memory: inhibition, shifting, and updating. Inhibition refers to the ability to suppress irrelevant or distracting information. As for the shifting mechanism it involves the flexibility to switch attention between tasks or stimuli. Working memory updating has been defined as the ability to dynamically modify the content of memory according to task requests [18, 19]. This model emphasizes that while these components are independent, they are interrelated and contribute to the shared variance of higher cognitive tasks such as problem-solving or complex decision-making processes.

Constraints concerning working memory are related to learning difficulties, which can be significant, but may be reduced by using personalized calibrated methods of support for children [20]. Working memory capacity and training in children with developmental disorders has received little attention from the research community, as opposed to children with a normative development [21]. Moreover, from the existing previous work, working memory has been associated differently to the various NDDs, (for a short overview of the different types of NDD, see the next Section “An overview…”).

Neurodevelopment disorders may be regarded as developmental deficits which lessen personal, social, academic and occupational functioning. Accordingly, the primary kinds of NDDs comprise attention-deficit/hyperactivity disorder, autism spectrum disorder, dyslexia, intellectual disability, among others. Some of the literature has shown that children with NDDs may benefit from game-based training [22, 23]. For instance, dyslexia has been associated to difficulties in verbal and visuospatial working memory [24], in phonological short-term memory and phonological awareness [25], whereas reading disabilities have been linked to phonological aspects of working memory and processes related to the central executive [26]. Children with developmental dyslexia have revealed deficits in verbal working memory, namely with a specific impairment in the phonological loop, whereas children with developmental coordination disorder have shown deficits in visuospatial working memory [27]. Moreover, children with specific language impairments have demonstrated difficulties regarding verbal working memory deficit and verbal short-term memory deficit [25]. In addition, children with specific arithmetic learning disabilities seem to have difficulty with phonological loop processes, visuo-spatial sketchpad processes and the central executive implicated in working memory [10]. Deficits in the central executive could lead children to have difficulties in inhibiting and updating information, as well as shifting attention from one task to another [10]. Regarding children with attention deficit disorders [25], William Syndrome [28] and coordination disorders [29], research has shown that there tends to be a difficulty concerning visuo-spatial working memory. Furthermore, children with Down Syndrome tend to have deficits in short-term memory [30], whereas those with autism spectrum disorder may show difficulties with central executive processes [31]. Also, comorbid children have shown poorer performance in verbal working memory, as well as in visuospatial working memory [27]. Considering that comorbidity (i.e. having two or more disorders) may be more frequent in children than previously thought, it is essential to provide a through diagnosis of the child’s condition to make decisions with regards assessment and competency training [31].

The literature has indicated that children with NDDs need training regarding their working memory capacity [32]. For instance, many children with autism spectrum disorder, intellectual disability and attention deficit/hyperactivity disorder have shown a low working memory capacity [32]. Moreover, those who have shown low working memory capacity have also revealed a low cognitive profile. Nonetheless, some children with autism spectrum disorder or attention deficit/hyperactivity disorder demonstrated higher working memory capacity than those previously mentioned. In fact, different NDDs have been associated with different patterns of working memory impairment [20]. This supports the notion that children with NDDs should have personalized training in terms of working memory capacity. As we are going to show in Sect. 2, the different types of NDD patients need not only different medications, but also different, and personalized therapy [1]. While serious games promised support for different health conditions, to tune these to support personalized NDD therapy, would require further research [2, 3].

## Serious games and children with NDDs

It is vital that working memory capacity be assessed and trained in children with NDDs with motivating resources, such as serious games because little to no digital tools are available to work with these children in a personalized way. Children with NDDs may have deficits that impact their academic performance [33]. The literature has revealed that over the last four decades, there has been an increase in the frequency of NDDs, and web-based interventions have the potential to improve results and decrease related symptoms which may hinder learning [33].

A recent meta-analysis on games to train executive functions revealed significant differences in working memory capacity [24] between children with NDDs who trained working memory with digital game-based training, and those who did not [1]. Moreover, game features had extra effects on training when training content was included. Also, digital game-based interventions revealed no significant differences between those who played at home and those who played in a laboratory setting. Overall, digital game-based interventions revealed training effects up to 9 weeks after implementation, providing opportunities for cognitive function rehabilitation [1]. Moreover, a recent review [34] found that active video-game-based interventions, where professionals were physically in the same room or in the same virtual platform at the same time as children, could improve children’s social behavior. Another review study mentioned that research is needed to assess the effectiveness of technology on a range of different NDDs [4], which we propose to do.

Previous research has indicated that serious games have the potential to train and improve working memory in children with NDDs in a ludic way within a school context, thus providing ecological validity [35]. Accordingly, serious games can inclusively increase divided attention skills, decrease distractibility, and help improve academic performance in reading and knowledge transfer. A systematic review and meta-analysis study revealed that cognitive interventions (e.g., with digital games) had a positive effect on children with NDDs’ working memory [36]. Nonetheless, more research and empirical evidence is needed to understand the full impact serious games can have on children with NDDs in terms of emotion recognition, anxiety reduction, stress regulation and rehabilitation [37]. In fact, clinical evidence is needed to understand the impact of serious games regarding the needs of children with NDDs [3, 4, 36].

This work exemplifies current possibilities for such games with functional prototypes supporting education. However, further study is needed to determine the validity of such games and connect the results to clinical aspects. Nonetheless, we propose to present reliability indicators of the serious game WorM with Item Response Theory (IRT). We question whether WorM constitutes a reliable measure to assess working memory and therefore, this paper focuses on the development and initial validity of the game.

The game WorM is one of the “EMPOWER games” included in a platform designed and developed by a European project (no 101060918, see https://project-empower.eu/# ). The games aim to utilize appropriate design elements for personalized educational intervention for students with NDDs. The interventions are developed by game designers in collaboration with psychologists.

## An overview of the most common NDDs in childhood

According to DSM-5, Neurodevelopmental Disorders (NDDs) [38] are considered to be thought disorders related to working memory, attention, and concentration. Developmental delay encompasses motor, intellectual, social-emotional, and speech development and can denote disabilities like cerebral palsy, Down syndrome, or autism. Intellectual disability in 1% of the population entails sub-average intellectual function and ability to take care of self, which requires special education and early intervention [39, 40]. Communication disorders, present in nearly one in ten children, result in speech and language impairment, with therapy and other forms of communication frequently required [39]. Autism spectrum disorder (ASD) [38] harms communication, social interaction, and behavior, and its characteristics include poor eye contact and repetitive behavior. Though it is lifelong, early intervention minimizes the outcome. ADHD [38] is expressed as inattention, hyperactivity, or a combination of both, which persists from childhood to adulthood. Though it cannot be cured, its symptoms can be managed by medication and therapy. DSM-5 redefined Specific Learning Disorder (SLD) in 2013 [38] to encompass reading, writing, and math challenges commonly linked with dyslexia (another NDD), affecting 20% of the population. SLD is not curable, but early treatment and accommodations help. Movement disorders [41, 42] intrude the brain and nervous system, interfering with voluntary and automatic movement, balance, and coordination and generally necessitating long-term supportive care.

## WorM: a working memory assessment and training game

### Initial game conceptualization

The game WorM was developed according to a sound theoretical background and according to the suggestions of experts who work with these children. The cognitive theory behind the construct we addressed as working memory in terms of updating in the game, is based on [37] executive functions model. Specifically, working memory/updating is one of the core components, along with shifting and inhibition. WorM was inspired by a standardized Corsi block computerized task that was developed by Milner [43], and as described by Macizo et al. [44], we added a concurrent task to the visual-spatial task. Concretely, we incorporated the insights provided by Macizo et al. [44], specifically in the development of phonological and visuospatial working memory tasks for children with NDDs.

While processing the spatial location and updating, children had to sort peppers that appeared on the screen according to a specific criterion. This addition increased the demands of the task, while not allowing for visual rehearsal strategies or visual fixations. A computerized task was conducted with children with autism spectrum disorder, ages 5–13 years, with a mean age of 8.55 years (*SD* = 2.3). In this study, the authors measured the number of trials correctly recalled up to the maximum block point. Scores were computed in percentages.

**Fig. 1:**
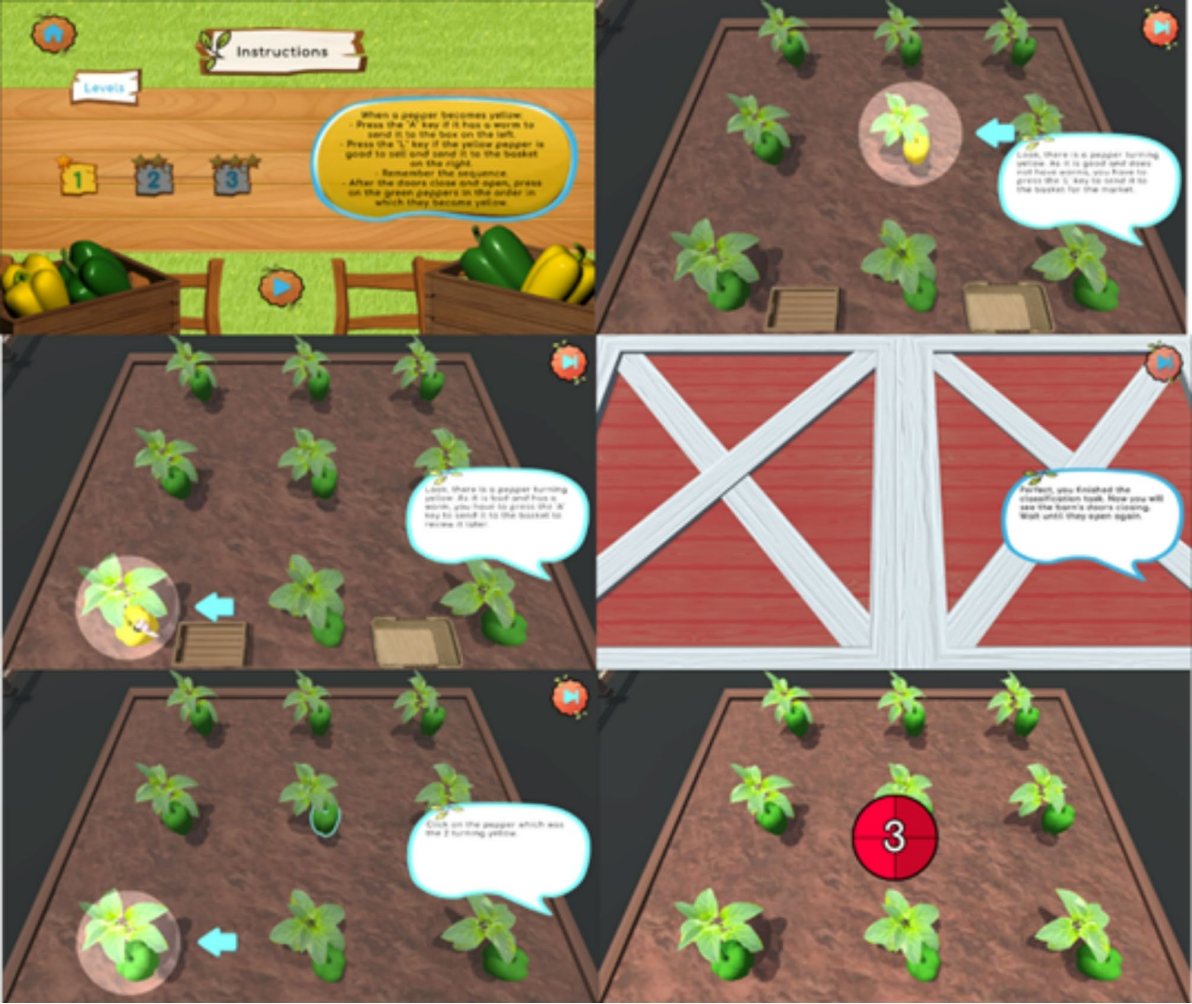
Instructions for players with examples of a ripe pepper, a pepper with a worm and a green pepper to choose from and then sort in the correct order of appearance

WorM was designed as a single player game with a sustainable theme that assesses and trains working memory in children with NDDs. The sustainable theme approaches eliminating food waste as a goal for the player in an Eco Farm. Children must sort ripe peppers from those that are not and those that have a worm so none are wasted. While doing this, they have a concurrent task. Also, they need to remember the sequence of locations from which they are supposed to pick up the ripe peppers (see Fig. 1). The child’s objective is to pick up yellow peppers as they are ripe, while considering the order. They tap the peppers. Also, they must also sort the ripe peppers from those that have a worm, by clicking on the left key for the good peppers that go into the crate and then to the market, and the right key for the peppers with a worm, that need to be salvaged, cut and cleaned. These peppers get transformed into pepper sauce or pickled peppers.

Specifically, children see 9 pepper plants that are in the vegetable garden (the lot is 16 × 16 cm). The stimuli that appear on the screen are a pepper plant with only one green pepper, a pepper plant with only one yellow pepper, and a pepper plant with only one yellow pepper that has a worm. The peppers change colors from green to yellow (1 s), gradually, one by one in random order. The pepper turns from green to yellow and back to the color from the start. The child sorts the peppers by clicking on the left and right keys. The plants with green peppers remain until 500 ms after the sequence was completed. Then a barn door is presented on the screen for 15 s. Afterwards, the screen with the pepper garden appears. On this screen the child touches each pepper plant in the order it turns yellow (ripens). For each sequence of 2–7, there are 5 trials per each sequence length. Times and task sequence followed Macizo et al.’s [44] instructions. Difficulty is used as a measure of feasibility by observing how children manage challenging tasks progressively. Incorrect answers (e.g., sorting mistakes) and performance trends provide insights into whether tasks are appropriately for the child’s ability. The difficulty level increases by increasing the number of peppers in the sequence: Level 1: 2 peppers (5 trials for 2 items); Level 2: 3 peppers (5 trials for 3 items); Level 3: 4 peppers (5 trials for 4 items). Level change occurs when children answer 3 out of 5 correctly from the previous sequence. In summary, WorM assesses working memory through the number of correct responses (e.g., correctly recalling sequences or sorting peppers accurately), also considering the difficulty level of the tasks, which adjust dynamically based on performance, thus, enabling children to train their working memory capacity according to their needs. In other words, the game is calibrated according to each child’s needs during gameplay.

The total correct trials can be computed. A correct trial is one in which the child recalls the yellow peppers in the correct order. Concurrently, the child must sort the items that have ripened, based on whether they have a worm or not. Subsequently, the answering accuracy is recorded.

### Scoring

Raw data were used to feed the algorithms which manage players’ scores. Specifically, we used children’s number of correct pepper classification, the correct selection and sorting of those peppers, as well as the longest sorted sequence of correct peppers sorted correctly. More information is available in the data analysis section of the user study.

## Study 1 - informing game conceptualization

### Participants and procedures

#### Ethical approval

was obtained for all three studies from the Ethics Committees of ISCTE-IUL and Babeș-Bolyai University. Group interviews with 4 to 6 participants each, were conducted to explore the perspectives of expert teachers and independent specialists regarding the usefulness and requirements of resources designed to enhance the executive functions of children with NDDs. Specifically, these interviews sought to extract insights into the essential features recommended by these experts for integration into the developmental phase of EMPOWER games. The interview script in appendix 1 also has a section on emotional skills and other cognitive dimensions and is part of a larger study, therefore the current study is restricted to working memory only which included questions 1, 2, and 3.

Participants were recruited based on specific criteria outlined by Etikan et al. [45], and the majority were selected due to their experience in working with children with NDDs. A total of 32 experts, comprising 29 females and 3 males, agreed to participate, with 19 being Portuguese nationals and 13 Romanian nationals. The participants included 14 teachers, 10 trained psychologists, 5 psycho-pedagogues, and 3 individuals with diverse training backgrounds.

Confidentiality and voluntary participation were emphasized, and all audio from the focus group interviews was recorded with participants’ consent. The data anonymization process occurred at this stage. Each interview, lasting approximately 60 min, took place online via Zoom, with participants being interviewed in their native language, either Portuguese or Romanian. Due to the confidential nature under the European Union data protection laws, all data is being stored in Donders Institute for Brain, Cognition, and Behaviour’s Repository at Radboud University. Once material has been published, data may be anonymously shared upon request.

### Measures – focus group interview script

We developed a semi-structured interview script specifically for this study (Appendix 1) comprising four distinct sections to elicit participants’ perspectives on the cognitive abilities of children with NDDs. Additionally, we sought insights into the tools currently employed by experts and identified areas where resources may be lacking in the assessment and promotion of cognitive competencies in primary school-aged children with NDDs.

The first section of the script included introductory and background questions, providing interviewees with information about the study, its relevance, and objectives. The second section focused on exploring cognitive factors and tools applicable to children with NDDs. The last section served to conclude the interviews, ensuring participants were informed of the session’s conclusion and reinforcing the commitment to ethical-deontological care throughout the process.

### Data analysis

Content analysis, following the methodology outlined by Braun and Clarke [46], was employed to analyze the focus group interviews with the NVivo software. This approach involved a detailed examination of the transcripts to discern common themes and patterns of significance relevant to our research questions. The six-step process proposed by Braun and Clarke [47] guided our thematic analysis, encompassing familiarization, coding, theme generation, theme review, definition and naming of themes, and the production of the final report.

The content analysis was conducted with an inductive approach due to the exploratory nature of this study. Themes were identified by reading the interviews, encompassing those derived from existing literature and those emerging directly from the collected data. These themes were then organized into codes. To enhance the reliability of our analysis, themes underwent adjustments through a process of data familiarization. To ensure further reliability, the involvement of two external judges, as suggested by Amado [48], was incorporated into the analysis process. The intraclass correlation revealed 100% agreement between the two judges. This agreement indicates that the judges consistently assigned the same codes to the themes under analysis without discrepancies, reflecting perfect inter-rater reliability.

### Results

**Fig. 2:**
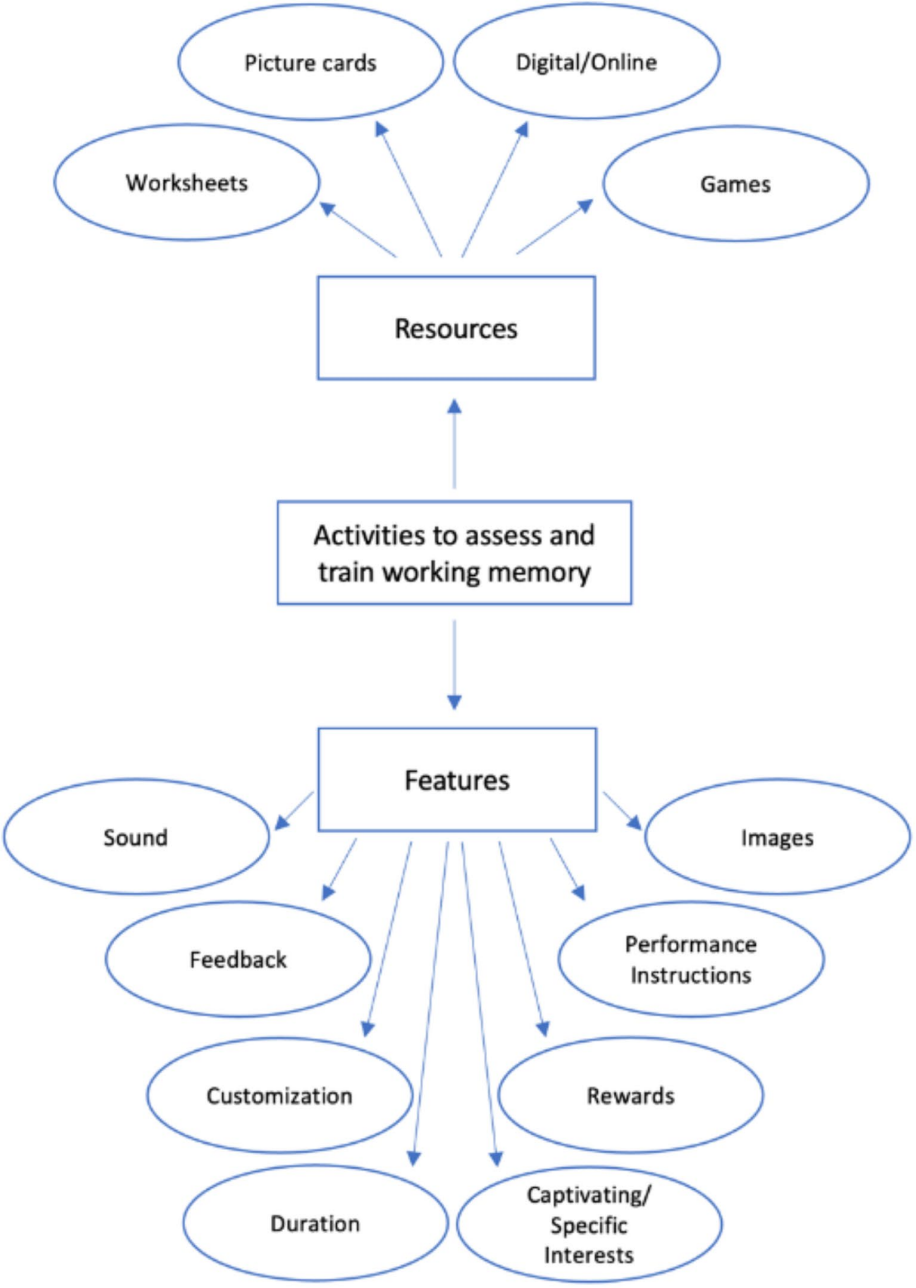
Experts’ perceptions of resources and features to inform game conceptualization

The purpose of the focus group interviews was to explore and gain a better understanding of the participants’ perspectives on the utilization and needs of resources in their field of expertise. The content analysis led to the development of a concept map, showing relevant themes and their respective categories [46]. Figure 2 depicts the topics related to the participants’ perceptions of activities they conduct with children to improve working memory assessment and training. Below are the identified themes and sub-themes from the content analysis, along with select examples from the experts’ discussions.

Regarding the resources the experts mentioned that they used when working with children with NDDs to assess and train working memory, the most prevalent were digital or online tasks (49,1%) “World Wall. It is used online (…)”, followed by games (19,3%) “We can turn a puzzle into a game” and worksheets (19,3%) “(…) spot the difference, using a pen and paper”and the least prevalent were Picture cards (12,3%) “(…) a game of naming pairs, in which they had to select the image that was the same”. This indicates that technological tools are commonplace in accessing and training working memory, and that besides being widely used, they are also motivational for children.

The specialists mentioned various features they deemed important when working with children with NDDs to assess and train working memory. Within the features, the most prevalent were captivating/specific interests (23%), these are the characteristics that are attractive to children, that appeal to their interests: “start with something playful, something attractive, something children love nowadays: the Pokémon, the Batman, or the pink world of unicorns, which many girls like, or it can even be just animals or themes that are important to work with them.” The second most prevalent was customization (22%), meaning that the game should be adapted to the needs of the child, being “Suitable for the child’s age and with tasks presented at increasing levels of difficulty”. The third most prevalent was the duration (13,4%) of the tasks, with mentions that “the tasks should be short”. With a lower prevalence, the experts mentioned rewards (10%) “In every activity I do, I always use positive reinforcement”, using images (8,5%) “(…) a game of naming pairs, in which they had to select the image that was the same “ and sound (8,5%) “Use the sound right or wrong”; “(…) captures their attention for the fact that it sounds good, it scratches their ear for music”, receiving feedback (8,5%) “Provide immediate feedback” and giving instructions (6,1%) “In terms of memory, I am thinking about the dictations given to them in some classes”.

### Discussion

In conclusion, Study 1 provided valuable insights to guide the conceptualization of the EMPOWER games aimed at enhancing working memory in children with neurodevelopmental disorders (NDDs). Through ethically approved focus group interviews with 32 experienced professionals—including teachers, psychologists, and psycho-pedagogues from Portugal and Romania—the study identified key features necessary for effective cognitive training tools. Thematic analysis revealed that digital tools are commonly used and well-received due to their motivational appeal. Experts emphasized the requirement to integrate those elements that match children’s specific interests, personalized material, and short task time. Additional factors such as reward, visual and auditory stimulation, simultaneous provision of feedback, and clear instructions were also emphasized as desirable. The findings name the feasibility and inclination of experts and form a reliable foundation for establishing motivating and operational working memory intervention in children with NDDs.

## Study 2 – validating game development

We performed a Focus Group interview with a Task (Appendix 2) to assess the face and content validity of the game. This interview script was also elaborated specifically for this study. The interview’s main objectives included:

1. To know the participants’ perceptions about integrating technological tools in the education process of children with NDDs.
2. To acquire information about the adequacy of the games’ characteristics.
3. To know the participants’ perceptions about the adequacy of the game (working memory) concerning cognitive competencies in children with NDDs.
4. To acquire information about the advantages and disadvantages regarding the use of technological games in training executive functions.
5. To identify possible problems when using the games with children with NDDs.
6. To acquire feedback about the usefulness and usability of the games.
7. To acquire feedback regarding most suitable end-users and how the feedback is provided to end-users.

### Participants

The participants of the focus group were 4 speech therapists and 8 special education teachers from the School Center for Inclusive Education Maria Montessori Constanta, Romania, a public institution that provides educational services to children with special needs. The mission “Maria Montessori” school is to encourage, support and improve the potential of children in partnership with their families to build independent life skills for an efficient social integration. The focus group was online, and every member had the opportunity to complete the reflection sheet individually and express their opinions.

### Resources

The resources used were a reflection sheet specifically designed for this study (Appendix 3), a focus-group interview script elaborated for this study and a tablet with the WorM game.

The focus-group interview script included the following questions:

1. What do you think about integrating technological tools in the education process of children with NDDs?
2. Have you ever used technology in your regular classes? Please give examples.
3. Please share with me your thoughts about the game and the notes you wrote down while playing the game.
4. Do you foresee any problems when assessing and training children with this game? If so, explain which.
5. Considering the children who you work with, which are the characteristics (what type of children, in terms of age, diagnosis, cognitive abilities) that we should consider when recruiting children in our studies and who you think can benefit most from the game?
6. How useful do you consider the game, in terms of assessing and training executive functions? What other types of cognitive processes and behavior do you believe that this training could help?
7. Do you think the game can predict these children’s academic performance, and/or social and emotional competencies?
8. How could you use the information extracted from the game in your everyday work?
9. What type of feedback do you want the game to produce?
10. How should feedback be given to the children through the game?
11. We are finishing our interview. Do you want to add something, or is there any other relevant aspect you want to address?
12. You may have access to general data from this first study. If you are interested, you can provide us with your email contact.

### Procedures

Interviewees were informed about the work to be carried out, the relevance of the study and its objectives. Interviewees’ collaboration was requested, and ethical guidelines and procedures adopted in the game’s development and future application, as well as anonymity of participants’ identity were communicated. Moreover, participants were informed of the confidentiality of the interview and data processing. The interview was recorded in audio with the authorization of the participants to gather information and to be destroyed after data analysis. Written consent to participate was given by participants.

Participants were first asked two general questions regarding the use of technology in the education of children with NDDs and case studies they may want to share. Participants were then given a reflection sheet and a tablet with the WorM game. Each participant had a reflection sheet to provide feedback to the interviewer. The group of interviewees then performed a task, which consisted of playing the game as a group and filling out the reflection sheet for a period of 10 min. After finishing, the focus group interview resumed with 6 questions to foster further group reflection regarding the task and specifically, concerning the game itself. Then, the interview was finalized with 3 final questions which fostered final comments from the participants and enabled the interviewer to thank them for their participation.

### Data analysis

In this paper, we present the results of a preliminary analysis of the validation session. NVivo software was used to investigate sub-categories of the main categories that emerged in the specialists’ discourse during the session.

### Results

The main aspects that were mentioned included the type of feedback given to participants, design features, such as colors, level differentiation, training for children. Specifically, the specialists mentioned that the WorM should provide visual feedback when sorting is done correctly and when it is wrong. They also referred to including pictures of the specific peppers on the baskets when collecting them so that it is easier for children to know which pepper goes in which basket. Since the game can be played on a tablet, but also on a computer, the specialists suggested having keys A and F from the keyboard for the children to respond to the stimuli. Moreover, the participants of the focus group also suggested having peppers of different colors, other than just yellow and green. In terms of the levels of the game, participants reported that the first level could have no worms in the peppers, including them only in levels 2 and 3. Furthermore, the participants suggested including a training session for children if they make 3 consecutive mistakes, as a reinforcement for them to keep playing. Also, the focus group participants considered that more visual feedback should be included in the game, especially when the answers are correct and wrong.

The participants also registered on their reflection sheet whether they disagreed, agreed more or less, or agreed with different statements regarding the game, namely:

- If the game reached its objective of assessing and training working memory in children with NDDs.
- If the range of difficulty was appropriate for children with NDDs.
- If the response time was adequate.
- If the colors were appropriate.

**Fig. 3:**
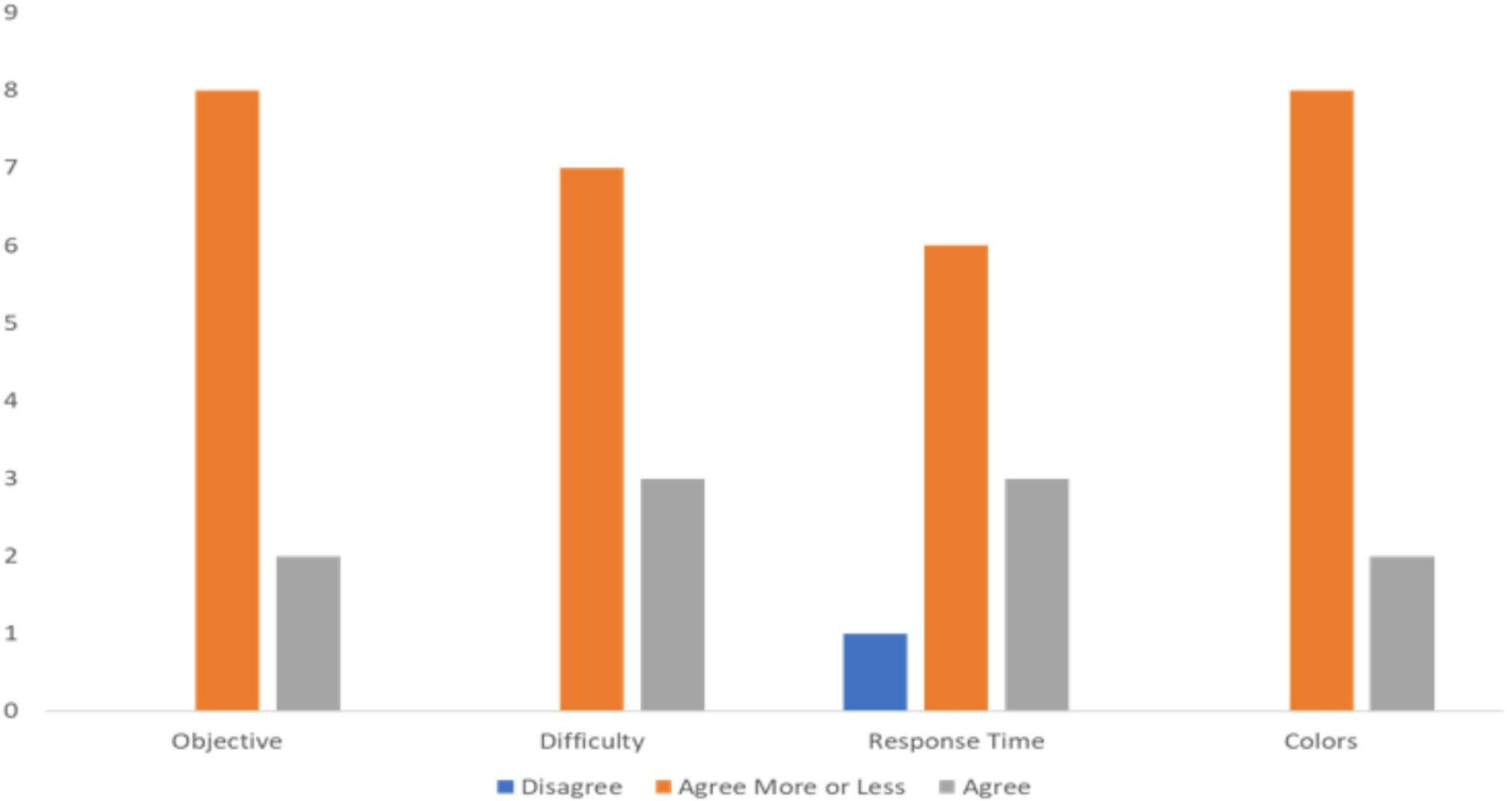
Results pertaining to the items of the reflection sheet

These results can be seen in Fig. 3.

Most participants agreed more or less, followed by those who agreed totally, and lastly, those to disagreed that the game reached its objectives (80%, 20%, 0% respectively), that the range of difficulty was appropriate (70%, 30%, 0% respectively), that the response time was adequate (60%, 30%, 10%, respectively) and that the colors were appropriate (80%, 20%, 0%, respectively). Based on the results obtained, we tried to clarify the objectives of the tasks, providing clearer and more objective instructions to reduce the perceived level of difficulty. There was also a concern to improve the definition of the images and the quality of the design and playability in the interaction between users and the game.

### Discussion

The focus groups from study 1 informed game development in Study 2. Specifically, study 1 played a crucial role in informing the conceptualization of the game by extracting expert insights on the essential features for enhancing executive functions in children with NDDs, which then guided Study 2 in validating the game’s development, ensuring its design, usability, and feedback mechanisms aligned with specialists’ recommendations. Both studies make relevant contributions to the development of gamified interfaces that enable the conception and design of games for the assessment and development of children with neurodevelopmental disorders. In particular, the development of gamified tasks must comply with a set of criteria that promote greater adaptability and personalization and engagement through greater interactivity [5]. The samples using therapists and teachers of children with special educational needs reinforced the importance of multimodal feedback that integrates multisensory feedback and a therapeutically relevant game that takes into account the very specific characteristics of this target audience [6]. However, one major limitation of these two studies is their small sample size, suggesting the need to move forward with a study 3 that incorporates a larger sample and allows quantitative information and infer conclusions about other psychometric dimensions relevant to the study.

## Study 3 – User pilot of worm

### Participants

Participants in our user study included 23 children with NDDs (Table 1) (*M*_*age*_ = 10.78, *SD* = 1.65, 69,6% male) from Romania and Portugal, as this game is being developed within the context of a European study and hence, we have data from these two countries. The sample was similar in terms of numbers, with 52% being Romanian.

**Table 1:** Item/person map of the CHEXI working memory variables and children’s reported working memory capacity by teachers

List of Neurodevelopmental Disorders		*N*
Bipolar disorder		9
Intellectual disability (moderate)		3
Attention deficit		2
Hyperactivity disorder		2
Learning disorder		3
Hyperkinetic disorder		3
Autism		2
Developmental delay		1
Polymorphous emotional disorder		1
Severe language delay		1
Selective mutism		1
ECI sequelae		1
Dyslexia		1
Dysphasia		1
School acquisition disorder		1

### Resources

A touch screen tablet with the WorM game was used (described above), along with a standardized task. The standardized task was the first subscale regarding children’s difficulty with working memory capacity (e.g., “Has difficulty remembering lengthy instructions.”) of the Child Executive Functioning Inventory (CHEXI), which includes 9 items. Special education teachers filled in this subscale regarding the children’s difficulty with working memory capacity on a Likert-type scale from 1 (definitely not true) to 5 (definitely true). The higher the score, the more difficulty with working memory capacity the child is perceived to have by their special education teacher or psychologist. This instrument has been validated for the Romanian and Portuguese child population with good psychometric properties [49].

### Procedures

All children, including their parents, gave consent to participate in this study and all ethical and deontological procedures were adopted according to the European Commission guidelines for ethical conduct and data protection in projects. Children played the WorM game individually with their special education teacher sitting next to them in a quiet room. Teachers filled in the subscale of the CHEXI regarding working memory.

### Data analysis

We aimed to investigate if these children encountered difficulty during gameplay and sought to confirm the reliability of the items incorporated in the serious game. Additionally, our objective was to explore their teachers’ assessment of their students’ working memory capacity and help improve this capacity. Following the approach of prior studies [50], we opted to compute IRT, which allowed us to gain a deeper understanding of the children’s performance in terms of their working memory capacity. Furthermore, Rasch analysis using Winsteps 3.9 was employed to assess the quality of the game’s tasks and to comprehend how their teachers rated the children’s working memory capacity. IRT has emerged as an alternative to the traditional approaches of classical test theory (CTT). Simply put, unlike CTT, whose statistics and item parameters are dependent on the characteristics of the tests and samples, IRT considers these two parameters independently. For example, it allows us to overcome a common error in psychometrics, which is that the greater the number of items, the greater the reliability of the scales and the smaller the number of items, the lower the reliability. Because in IRT participants and items are calibrated on the same scale, it makes it easier to interpret the items assessed. Unlike CTT, in which values tend to be biased in favor of mean values, the logit measures used in IRT do not assume uniformity of values across all the items in a scale [51]. The rationale behind IRT lies in its ability to assess the likelihood of a person with a particular ability succeeding on a specific item, considering its level of difficulty. This contrasts with the classic approach to testing which uses percentile scales that consider performance in relation to the sample group [51].

This analysis facilitated the estimation of the children’s and their respective teachers’ scores on a one-dimensional logit scale, enabling an evaluation of the properties of each task embedded in the game, as well as in the standardized task’s items, respectively. The Rasch polytomous methodology was applied, respectively to examine the children’s and teachers’ ratings. The Partial Credit Model (PCM), an extension of the Rasch model for polytomous items [52], was employed for linear measures of observations on ordinal scales. The PCM formula for this study is log(Pnik/Pni(k1))/ Hn bitki, where Pnik represents the probability of person n responding in category k when encountering item i. Furthermore, Pni(k1) is the probability of the response being in category k1, whereas Hn signifies an individual’s ability, bi denotes the difficulty (or, as framed in this study, children’s working memory capacity and teachers’ level of rating, respectively) of item i, and tki is the step calibration in the rating scale threshold, defined as the position equivalent to the equal probability of responses in adjacent categories k1 and k [53].

In this study, each working memory task/trial was based on the number of correct items and the teachers’ ratings were assessed on a continuous scale, as mentioned in the game description and resources section, respectively. A higher score in the game indicated greater working memory capacity, whereas a lower score indicated lower working memory capacity. Conversely, a higher score in the standardized task (5) indicated more difficulty with working memory capacity, whereas a lower score indicated less difficulty (1).

All tasks in the game and all items from the standardized task were examined to determine their fit to the model (*p* <.01) and identify tasks/items with extreme infit and outfit mean square residuals. We examine whether tasks/items have infit standardized mean squares lower than 1.5 and outfit standardized mean squares lower than 2.0, as recommended in the literature [54]. Infit refers to a form of fit assessment that considers inlier sensitivity and information weighting. It demonstrates increased sensitivity to the response patterns associated with targeted items for individuals, and vice versa. For instance, infit assessments may indicate overfitting in the context of Guttman patterns and underfitting in alternative curricula or specific clinical groups with idiosyncrasies. Identifying and addressing these patterns can pose challenges in terms of diagnosis and resolution. Outfit denotes a fit assessment that is sensitive to outliers. It places greater emphasis on responses to items with difficulty levels significantly distant from an individual, and vice versa. For instance, outfit evaluations may signal overfitting in the context of imputed responses, and underfitting in cases of fortunate guesses or careless mistakes. Typically, these issues are straightforward to identify and address [55].

### Results

We used an IRT approach to investigate children’s working memory capacity during gameplay. The aim was to assess whether children encountered difficulties with the tasks/trials presented in the game and to evaluate the reliability of each of those tasks. We proposed to do the same with the standardized task which was filled in by their teachers with regards to their students.

The Correct Pepper Classification tasks in level 1 showed infit/outfit scores exceeding 1.5, and level 3 showed infit surpassing 1.5, whereas none of the standardized task variables of the CHEXI working memory scale displayed infit/outfit scores exceeding 1.5 or z statistics surpassing 2.0, respectively, except for item 9 (“Has difficulty remembering lengthy instructions.”) [55]. The most difficult task/trial of WorM was the Correct Pepper Classification with Incorrect Sorting task in level 1, with a reported/difficulty level of 1.58log, whereas the Correct Pepper Classification task in level 3 was the easiest, registering a reported/difficulty level of -2.17log. Consequently, the distribution indicated a wide range of difficulty (1.58 < Di < -2.17): **Level 1** (2 peppers, 5 trials) as an introductory level with minimal cognitive load, **Level 2** (3 peppers, 5 trials) as an intermediate difficulty level with added cognitive challenges, and **Level 3** (4 peppers, 5 trials) as the most advanced level, requiring increased working memory abilities. Hence forth, the varying levels of difficulty were analysed, allowing us to examine how children progressed through increasingly complex working memory challenges, to provide a dynamic and adaptive game level structure.

**Fig. 4:**
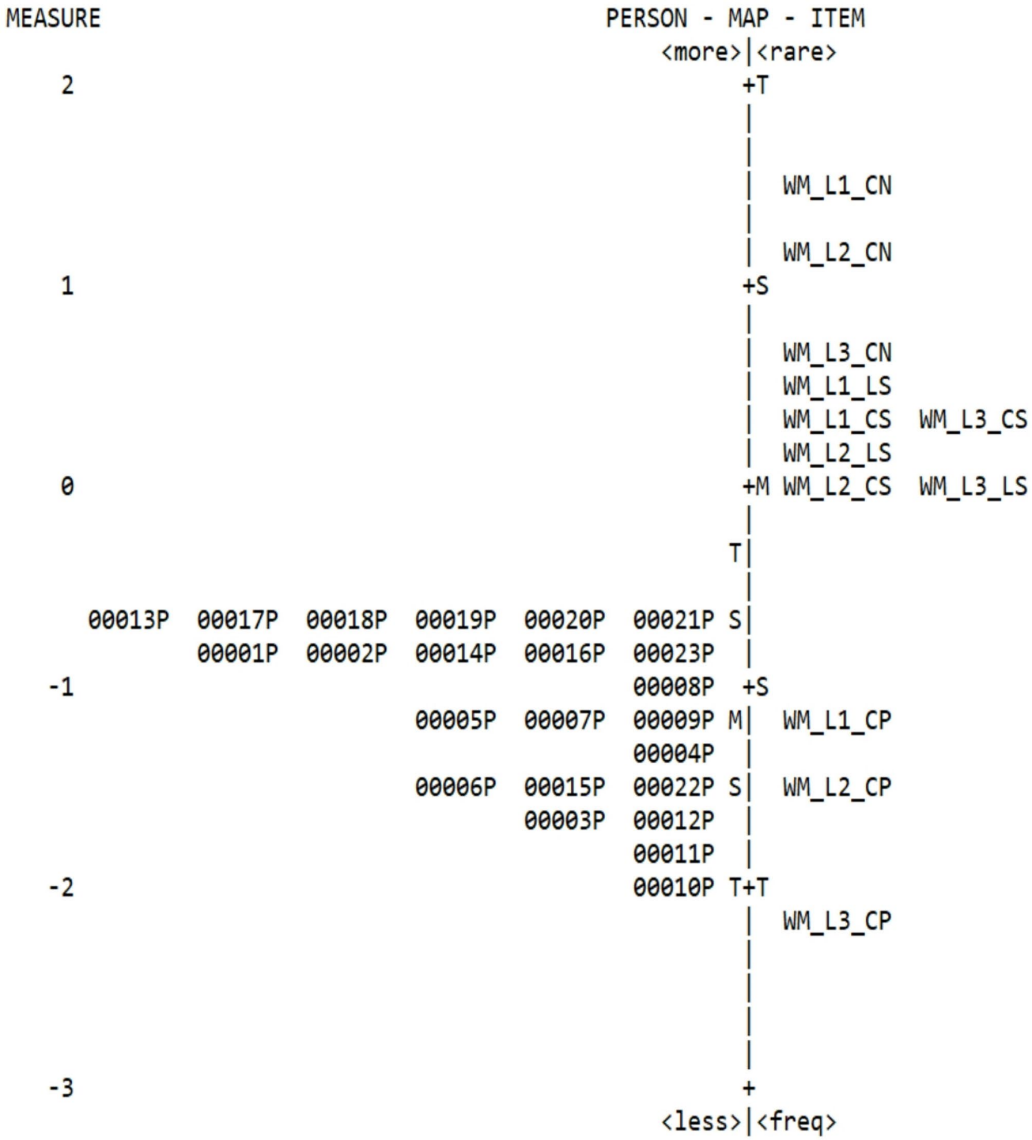
Item/person map of the WorM variables and children’s working memory capacity during gameplay

Furthermore, child 10 (aged 12 female diagnosed with moderate intellectual disability and hyperkinetic disorder) revealed more difficulty in terms of working memory capacity during gameplay, whereas children 13, 18, 20 and 21 (all females with bipolar disorder aged 8, 13, 12 and 12, respectively) revealed less (Fig. 4).

The analysis of the CHEXI working memory standardized task revealed that the most difficult item to report was 19 (“Has difficulty understanding verbal instructions unless he/she is also shown how to do something.”), with a reported/difficulty level of 1.50log, whereas item 7 (“Has difficulty coming up with a different way of solving a problem when he/she gets stuck.”) was the easiest to report with a score of -1.14log. Furthermore, the results of children’s reported working memory capacity by teachers revealed that children 2, 8, 10, 12 and 20 had infit values above 1.5 and, and child 20 also had an outfit score and z statistics above 2.0 [56].

**Fig. 5:**
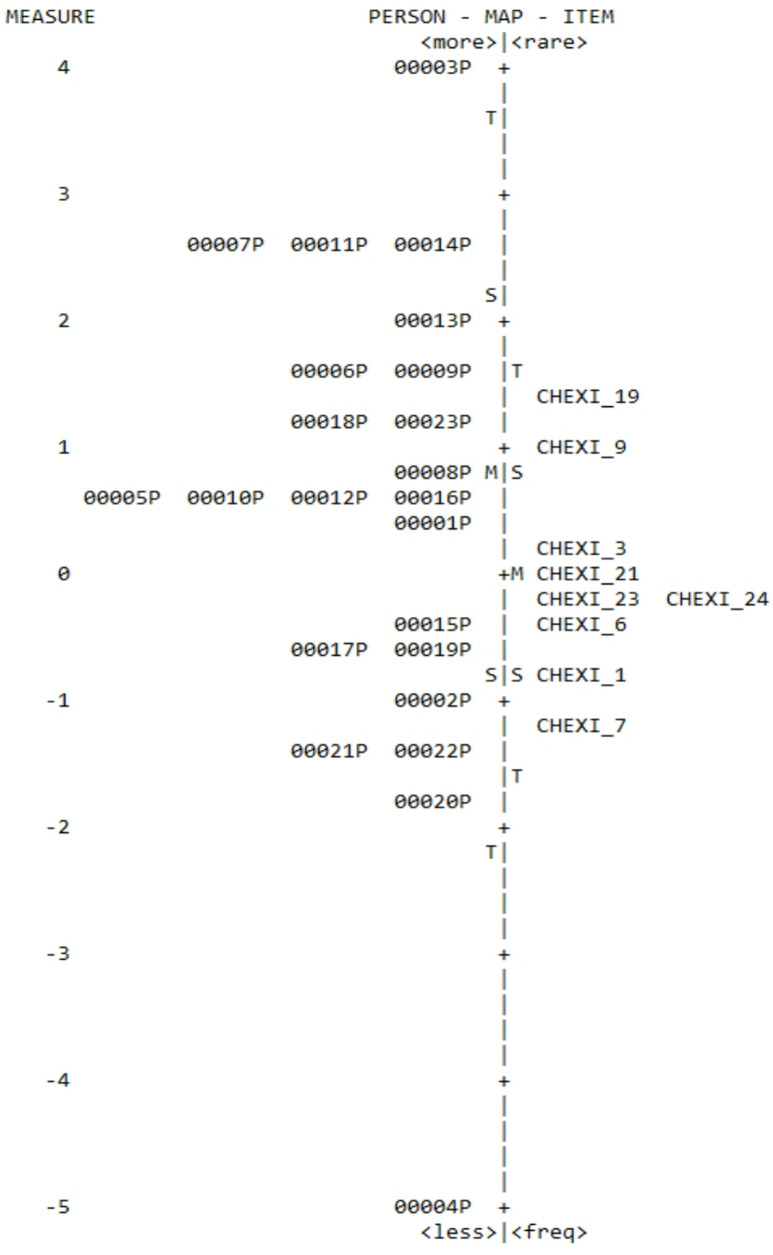
Item/person map of the CHEXI working memory variables and children’s reported working memory capacity by teachers

Concerning difficulty in reporting children’s working memory capacity, child 3 (aged 9 male diagnosed with moderate intellectual disability and hyperkinetic disorder) was the most difficult to report on with a level of 3.94log, whereas child 20 (aged 12 female with bipolar disorder) was the easiest to report on with a score of -1.77log. The distribution illustrated an adequate range of difficulty in the standardized task (1.50 < Di < -1.14) (Fig. 5).

We employed additional reliability indicators from the Rasch model for working memory capacity during gameplay and children’s reported working memory capacity by their teachers, such as Person Separation Reliability (PSR) and Item Separation Reliability (ISR). The PSR, was 0.84 for children’s working memory capacity during gameplay, and 0.91 for children’s reported working memory capacity by their teachers. The ISR for children’s working memory capacity during gameplay was 0.97, and 0.83 for children’s reported working memory capacity by their teachers, indicating good internal consistency/reliability of the tasks/trials in the WorM game and in the CHEXI items from the scale on working memory [57]. Furthermore, WorM revealed a Cronbach’s alpha of 0.73 and the working memory CHEXI scale showed 0.92. Hence, both instruments performed well with the sample presented in this study.

This study was conducted within the context of a European initiative, focusing on Romania and Portugal to address cross-cultural applicability and regional differences in educational practices and neurodevelopmental support systems. By utilizing advanced psychometric approaches such as IRT and Rasch analysis, we gained nuanced insights into the working memory capacities of children with NDDs, surpassing traditional methods that often fail to account for individual variability. Notably, the differences in task difficulty within the WorM game highlight the importance of tailoring game design to balance engagement and challenge, particularly for children with varying cognitive profiles. The findings have practical implications for serious game development, advocating for iterative design processes informed by empirical data. Furthermore, the cross-cultural lens of this study underscores the need to explore how local educational practices shape cognitive outcomes, offering a foundation for policy recommendations aimed at improving support for children with NDDs. Future research could expand on these findings by incorporating larger, more diverse samples and longitudinal designs to track the impact of serious games on cognitive development over time. By addressing these areas, we aim to contribute to the growing body of evidence supporting serious games as effective tools for enhancing cognitive abilities in children with NDDs.

## General discussion

Empowering children with motivating resources, such as serious games, may promote their development as independent learners who can identify and fulfill their own learning needs [5]. WorM offers reliable assessment and a promising training approach regarding working memory in children with NDD’s. Our results from the user study revealed that children with hyperkinetic disorder may have more difficulty in responding of the game due to physical limitations, although more research is recommended to attest this finding with a larger sample with such characteristics. Nonetheless, the WorM game revealed good reliability indicators (both on the game and participants’ side), along with a standardized task, even though it showed a wider range of difficulty than the standardized task. Therefore, cognitive training where individuals engage in gamified repetitive exercises or activities involving the repetition of standardized cognitive tasks may foster neuroplasticity, encouraging the brain to adapt and enhance its cognitive functions over time through repeated and focused exercises [58].

In this study, PCM (partial credit model) was used to assess the difficulty of each task/trial within the game and the standardized measure, allowing us to derive linear measures from ordinal data. Specifically, each level in the WorM game was structured to progressively increase in cognitive load: Level 1 (2 peppers, 5 trials) was designed as an introductory stage with minimal working memory demands, Level 2 (3 peppers, 5 trials) presented intermediate challenges, and Level 3 (4 peppers, 5 trials) required advanced working memory skills. These levels provided a structured framework for assessing children’s cognitive abilities, with PCM modelling allowing us to capture the probability of success at each level and trial.

The results confirmed variations in task difficulty, with the most challenging task being the Correct Pepper Classification with Incorrect Sorting task in Level 1 (1.58 log), while the easiest was the Correct Pepper Classification task in Level 3 (-2.17 log). By incorporating PCM, we were able to evaluate whether the response patterns aligned with expected difficulty hierarchies and whether specific tasks or trials exhibited misfitting response patterns. Moreover, when applying PCM to the standardized task (CHEXI working memory scale), we found that most items conformed to the model’s expectations, except for item 9 (“Has difficulty remembering lengthy instructions”). The application of PCM enabled a direct comparison between children’s working memory performance in a game-based environment and their teacher-reported working memory abilities.

Further applications of WorM for assessment and training of children with NDDs are needed in randomized controlled trials, nonetheless. Moreover, future research could use WorM with other games to measure different executive functions and emotions involved in game-playing. Accordingly, future studies could consider the role of individual differences, as well as children’s visual-motor coordination to acquire a better understanding of their effect on the efficiency of the game without ongoing user-training [57]. Resources, such as the WorM game, need experts to manage them and overcome obstacles which may arise during applications, such as children’s unexpected behavior, reactive responses of special education teachers which compromise data gathering, high error percentage due to poor algorithm calibration, low signal strength, and low data transfer rate [11, 59]. Moreover, the WorM game’s accuracy in assessing and training working memory, as well as acquiring results, may be reduced due to the difficulty children with NDDs experience in maintaining exact mental states during different moments in the session, as it may lead to cognitive fatigue and consequent demotivation [5]. Nonetheless, the WorM prototype will continue to be developed according to the specialists’ suggestions so that it may be functional for professionals and children with NDDs.

### Limitations

This study is not without limitations. Results from the user study should be interpreted with caution and as an exploratory approach to assessing working memory in children with neurodevelopmental disorders, especially due to the small sample size. Future studies should invest in testing this game using longitudinal studies with a larger sample size, focusing on specific NDDs. Moreover, data was gathered from only one session per child. Future studies should focus on ensuring that children get more practice through more sessions so that they have further opportunities to develop their working memory capacity. The fact that children could play for more than one session could also provide a more detailed account of children’s working memory capacity, considering their daily social and physical context. There are limitations related to cognitive training interventions that should not be neglected. We highlight the difficulty in transferring the specificities of the game and the tasks associated with the game to the classroom context and external reality. Gains can be associated with performance in the game and not so much with the development of cognitive skills. The question of the impact of cognitive training in cognitive gamified games is still very unclear and deserves further development. Finally, each participant’s entry profile is very different, and conditions the approach to the game (motivations), which can have a different impact on the cognitive effects of participating in this type of task. Although the main objective of this investigation was not to pursue longitudinal tendencies of working memory capacity, we propose that future studies could invest in more complete and complex (with dynamic and personalized algorithms) longitudinal analyses, such as time series analyses and multilevel modelling. Lastly, we did not have specific information about the mean age and years of experience. Future studies could provide this information in future studies.

## Conclusion

In summary, the development and evaluation of the WorM game — a digital intervention designed to assess and enhance working memory in children with NDDs — demonstrated a thoughtful, evidence-based progression through three interconnected studies. Study 1 gathered foundational insights from experts on desirable game features, emphasizing engagement, personalization, and multimodal feedback. These findings directly informed Study 2, which validated the game’s design through expert reviews and iterative improvements. Finally, Study 3 piloted the game with children, using both behavioral performance and standardized assessments to evaluate its effectiveness and task quality. Despite a limited sample size, the overall results show strong alignment between expert expectations and children’s engagement, pointing to the game’s potential as a valid and motivating tool for working memory support. Together, these studies offer a promising take-home message: with thoughtful design, ethical rigor, and collaboration between specialists and educators, serious games like WorM can become powerful, inclusive tools to support cognitive development in diverse educational settings.

## Electronic supplementary material

Below is the link to the electronic supplementary material.

Supplementary Material 1

Supplementary Material 2

Supplementary Material 3

## Data Availability

Due to the confidential nature under the European Union data protection laws, data is being stored in Donders Institute for Brain, Cognition, and Behaviour’s Repository at Radboud University. Once material has been published, data may be anonymously shared upon request.

## Abbreviations

NDD: Neurodevelopmental disorder
ASD: Autism spectrum disorder
ADHD: Attention deficit hyperactivity disorder
SLD: Specific Learning Disorder
DSM5: Diagnostic and statistical manual of mental disorders: 5^TH^ Edition

## References

[1] Ren X, Wu Q, Cui N, Zhao J, Bi H-Y. Effectiveness of digital game-based trainings in children with neurodevelopmental disorders: A meta-analysis. Res Dev Disabil. 2023;133:104418. 10.1016/j.ridd.2022.104418. 36603312 10.1016/j.ridd.2022.104418

[2] Roording-Ragetlie S, Klip H, Buitelaar J, Slaats-Willemse D. Working memory training in children with neurodevelopmental disorders. Psychology. 2016;7(3):310–25. 10.4236/psych.2016.73034.

[3] Kerns KA, Macoun S, MacSween J, Pei J, Hutchison M. Attention and working memory training: A feasibility study in children with neurodevelopmental disorders. Appl Neuropsychol Child. 2017;6(2):120–37. 10.1080/21622965.2015.1109513. 27049769 10.1080/21622965.2015.1109513

[4] Valentine AZ, Brown BJ, Groom MJ, Young E, Hollis C, Hall CL. A systematic review evaluating the implementation of technologies to assess, monitor and treat neurodevelopmental disorders: A map of the current evidence. Clin Psychol Rev. 2020;80:101870. 10.1016/j.cpr.2020.101870. 32712216 10.1016/j.cpr.2020.101870

[5] Papanastasiou G, Drigas A, Skianis C, Lytras M. Brain computer interface based applications for training and rehabilitation of students with neurodevelopmental disorders. A literature review. Heliyon. 2020;6(9). 10.1016/j.heliyon.2020.e04250. 10.1016/j.heliyon.2020.e04250 PMC7482019 32954024

[6] Alamdari N, Haider A, Arefin R, Verma AK, Tavakolian K, Fazel-Rezai R. A review of methods and applications of brain computer interface systems. IEEE International Conference on Electro Information Technology. 2016;2016:345–50. 10.1109/EIT.2016.7535263

[7] van Balkom TD, Berendse HW, van der Werf YD, Vriend C. Feasibility of a cognitive training game in Parkinson’s disease: the randomized parkin’play study. Neuropsychobiology. 2020;79(4):270–8. 10.1159/000509685. 32756067 10.1159/000509685 PMC7592931

[8] Lawrence BJ, Gasson N, Bucks RS, Loftus AM. Computerized cognitive training in Parkinson’s disease: A systematic review and meta-analysis. Neuroscience & Biobehavioral Reviews. 2022.;137,104627. 10.1016/j.arr.2022.101671 10.1016/j.arr.2022.101671 35714854

[9] Wang P, Fang Y, Qi JY, Li HJ, Fisherman. A serious game for executive function assessment of older adults. Assess. 2023;30(5):1499–513. 10.1177/10731911221105648. 10.1177/10731911221105648 35762827

[10] Passolunghi MC. Working memory and arithmetic learning disability. Working memory and neurodevelopmental disorders. New York: Psychology; 2006. pp. 113–38.

[11] Rao PA, Beidel DC, Murray MJ. Social skills interventions for children with Asperger’s syndrome or high-functioning autism: A review and recommendations. J Autism Dev Disord. 2008;38(2):353–61. 10.1007/s10803-007-0402-4. 17641962 10.1007/s10803-007-0402-4

[12] Barrett LF, Bliss-Moreau E, Quigley KS, Aronson KR. Interoceptive sensitivity and self-reports of emotional experience. J Pers Soc Psychol. 2004;87(5):684–97. 10.1037/0022-3514.87.5.684. 15535779 10.1037/0022-3514.87.5.684 PMC1224728

[13] Miyake A, Friedman NP. The nature and organization of individual differences in executive functions: four general conclusions. Curr Dir Psychol Sci. 2012;21(1):8–14. 10.1177/0963721411429458. 22773897 10.1177/0963721411429458 PMC3388901

[14] Baddeley D, Hitch G, Allen R. A Multicomponent Model of Working memory. In: Baddeley D, Hitch G, editors. Working memory. Oxford: Oxford University Press; 2020. pp. 10–43. 10.1093/oso/9780198842286.003.0002.

[15] Baddeley D, Hitch G. Working memory. In: Bower GH, editor. Psychology of learning and motivation. Volume 8. New York: Academic; 1974. pp. 47–89. 10.1016/S0079-7421(08)60452-1.

[16] Daneman M, Carpenter PA. Individual differences in working memory and reading. J Verbal Learn Verbal Behav. 1980;19(4):450–66. 10.1016/S0022-5371(80)90312-6.

[17] Miyake A, Shah P. Models of working memory: toward unified theories of working memory: emerging general consensus, unresolved theoretical issues, and future research directions. Cambridge: Cambridge University Press; 1999.

[18] Morris N, Jones DM. Memory updating in working memory: the role of the central executive. Br J Psychol. 1990;81:111–21. 10.1111/j.2044-8295.1990.tb02349.x.

[19] Carretti A, Borella E, De Beni R. Does strategic memory training improve the working memory performance of younger and older adults? Exp Psychol. 2007;54(4):311–20. 10.1027/1618-3169.54.4.311. 17953152 10.1027/1618-3169.54.4.311

[20] Gathercole SE, Alloway TP. Practitioner review: Short-term and working memory impairments in neurodevelopmental disorders: diagnosis and remedial support. J Child Psychol Psychiatry. 2006;47(1):4–15. 10.1111/j.1469-7610.2005.01446.x. 16405635 10.1111/j.1469-7610.2005.01446.x

[21] Alloway TP, Gathercole SE, Pickering SJ. Verbal and visuospatial Short-Term and working memory in children: are they separable?? Child Dev. 2006;77(6):1698–716. 10.1111/j.1467-8624.2006.00968.x. 17107455 10.1111/j.1467-8624.2006.00968.x

[22] Bertoni S. Tesi redatta con il contributo finanziario della Fondazione CARIPARO. 2019;1–87.

[23] Cheng LH, Liu YW, Wu CC, Wang S, Tsai YC. Psychobiotics in mental health, neurodegenerative and neurodevelopmental disorders. J Food Drug Anal. 2019;27(3):632–48. 10.1016/j.jfda.2019.01.002. 31324280 10.1016/j.jfda.2019.01.002 PMC9307042

[24] Pickering SJ. Working memory in dyslexia. Working memory and neurodevelopmental disorders. New York: Psychology; 2006. pp. 7–40.

[25] Archibald LM, Gathercole SE. Short-term and working memory in specific Language impairment. Int J Lang Commun Disord. 2006;41(6):675–93. 10.1080/13682820500442602. 17079222 10.1080/13682820500442602

[26] Swanson HL. Working memory and reading disabilities: both phonological and executive processing deficits are important. Working memory and neurodevelopmental disorders. New York: Psychology; 2006. pp. 59–88.

[27] Maziero S, Tallet J, Bellocchi S, Jover M, Chaix Y, Jucla M. Influence of comorbidity on working memory profile in dyslexia and developmental coordination disorder. J Clin Exp Neuropsychol. 2020;42(7):660–74. 10.1080/13803395.2020.1798880. 32746703 10.1080/13803395.2020.1798880

[28] Rowe ML, Mervis CB. Working memory in Williams syndrome. Working memory and neurodevelopmental disorders. New York: Psychology; 2006. pp. 267–93.

[29] Alloway TP. Working memory skills in children with developmental coordination disorder. Working memory and neurodevelopmental disorders. New York, NY: Psychology; 2006. pp. 161–85.

[30] Belleville S, Ménard É, Mottron L, Ménard M-C. Working memory in autism. Working memory and neurodevelopmental disorders. New York, NY: Psychology; 2006. pp. 213–38.

[31] Morris-Rosendahl AJ, Crocq MA. Neurodevelopmental disorders—the history and future of a diagnostic concept. Dialogues Clin Neurosci. 2020;22(1):65–72. 10.31887/DCNS.2020.22.1/macrocq. 32699506 10.31887/DCNS.2020.22.1/macrocq PMC7365295

[32] Márquez-Caraveo M, et al. Children and adolescents with neurodevelopmental disorders show cognitive heterogeneity and require a person-centered approach. Sci Rep. 2021;11(1). 10.1038/s41598-021-97551-6. 10.1038/s41598-021-97551-6 PMC8445997 34531454

[33] Khan K, Hall CL, Davies EB, Hollis C, Glazebrook C. The effectiveness of web-based interventions delivered to children and young people with neurodevelopmental disorders: systematic review and meta-analysis. J Med Internet Res. 2019;21(11). 10.2196/13478. 10.2196/13478 PMC6858614 31682573

[34] Rafiei Milajerdi H, Dewey D. Is active video gaming associated with improvements in social behaviors in children with neurodevelopmental disorders: a systematic review. Child Neuropsychol. 2022. 10.1080/09297049.2022.2046721. 35236234 10.1080/09297049.2022.2046721

[35] Kerns AM, Renno P, Kendall PC, Wood JJ, Storch EA. Anxiety disorders interview Schedule–Autism addendum: reliability and validity in children with autism spectrum disorder. J Clin Child Adolesc Psychol. 2017;46(1):88–100. 10.1080/15374416.2016.1233501. 27925775 10.1080/15374416.2016.1233501 PMC5441235

[36] Robinson KE, Kaizar E, Catroppa C, Godfrey C, Yeates KO. Systematic review and meta-analysis of cognitive interventions for children with central nervous system disorders and neurodevelopmental disorders. J Pediatr Psychol. 2014;39(8):846–65. 10.1093/jpepsy/jsu031. 24864276 10.1093/jpepsy/jsu031

[37] Miyake A, Friedman NP, Emerson MJ, Witzki AH, Howerter A, Wager TD. The unity and diversity of executive functions and their contributions to complex ‘frontal lobe’ tasks: A latent variable analysis. Cogn Psychol. 2000;41(1):49–100. 10.1006/cogp.1999.0734. 10945922 10.1006/cogp.1999.0734

[38] American Psychiatric Association. Diagnostic and statistical manual of mental disorders: DSM-5TM. 5th ed. Arlington, VA: American Psychiatric Publishing, Inc.; 2013. 10.1176/appi.books.9780890425596.

[39] Morin A. What you need to know about Developmental Delays. Understood. 2014. Available from: https://www.understood.org/en/learning-attention-issues/treatments-approaches/early-intervention/what-you-need-to-know-about-developmental-delays

[40] Gupta SN, Gupta VS, Ahmed A. Common developmental delay in Full-term children: A common neurological profile to aid in clinical diagnosis. J Clin Dev Biol. 2016;3(2).

[41] Scahill L, Specht M, Page C. The prevalence of tic disorders and clinical characteristics in children. J Obsessive-Compulsive Relat Disord. [In press]. 10.1016/j.jocrd.2014.06.002 PMC4243175 25436183

[42] Bitsko RH, Holbrook JR, Visser SN, Mink JW, Zinner SH, Ghandour RM, Blumberg SJ. A National profile of tourette syndrome. J Dev Behav Pediatr. 2014;35(1):17–22. 10.1097/DBP.0000000000000065 PMC4484726 24906033

[43] Milner B. Interhemispheric differences in the localization of psychological processes in man. Br Med Bull. 1971;27(3):272–7. 10.1093/oxfordjournals.bmb.a070866. 4937273 10.1093/oxfordjournals.bmb.a070866

[44] Macizo P, Soriano M, Paredes N. Phonological and visuospatial working memory in autism spectrum disorders. J Autism Dev Disord. 2016;46(9):2956–67. 10.1007/s10803-016-2835-0. 27314268 10.1007/s10803-016-2835-0

[45] Etikan I, Musa SA, Alkassim RS. Comparison of convenience sampling and purposive sampling. Am J Theor Appl Stat. 2016;5(1):1–4. 10.11648/j.ajtas.20160501.11.

[46] Braun V, Clarke V. Using thematic analysis in psychology. Qual Res Psychol. 2006;3(2):77–101. 10.1191/1478088706qp063o.

[47] Braun V, Clarke V. Successful qualitative research: A practical guide for beginners. 1st ed. London: Sage; 2013.

[48] Amado I. Manual de Investigação Qualitativa em Educação. 3rd ed. 2017. 10.14195/978-989-26-1390-1

[49] Catale A, Meulemans T, Thorell LB. The childhood executive function inventory. J Atten Disord. 2013;19(6):489–95. 10.1177/1087054712470971. 23355496 10.1177/1087054712470971

[50] Francisco SM, Veiga Simão AM, Ferreira PC, Martins MJ. Das D. Cyberbullying: the hidden side of college students. Comput Hum Behav. 2015;43:167–82. 10.1016/j.chb.2014.10.045.

[51] Ferreira AI, Almeida LS, Prieto G. The role of processes and contents in human memory: an item response theory approach. J Cogn Psychol. 2011;23(7):873–85. 10.1080/20445911.2011.584692.

[52] Rasch G. Probabilistic Models for Some Intelligence and Attainment Tests. 1980.

[53] Wright BD, Masters GN. Rating scale analysis: Rasch measurement. Chicago: Mesa; 1982.

[54] Bond TG, Fox CM. Applying the Rasch model. Psychology; 2013.

[55] Linacre M. What do infit and outfit, Mean-square and standardized mean? Rasch Meas Trans. 2002;16(2):878.

[56] Fox M, Jones JA. Uses of Rasch modeling in counseling psychology research. J Couns Psychol. 1998;45(1):30–45. 10.1037/0022-0167.45.1.30.

[57] Hammer GP, Du Prel JB, Blettner M. Vermeidung verzerrter ergebnisse in beobachtungsstudien: teil 8 der Serie Zur bewertung wissenschaftlicher publikationen. Dtsch Arztebl. 2009;106(41):664–8. 10.3238/arztebl.2009.0664.

[58] Guglietti B, Hobbs D, Collins-Praino LE. Optimizing cognitive training for the treatment of cognitive dysfunction in Parkinson’s disease: current limitations and future directions. Front Aging Neurosci. 2021;13. 10.3389/fnagi.2021.709484. 10.3389/fnagi.2021.709484 PMC8549481 34720988

[59] Ramadan RA, Refat S, Elshahed MA, Ali RA. Basics of brain computer interface. Intell Syst Ref Libr. 2015;74:31–50. 10.1007/978-3-319-10978-7_2.

