# Supplementary material for: Neurodevelopmental disorders: assessing and training working memory
Source: BMC Psychol. 2025 Oct 21;13:1163. doi: 10.1186/s40359-025-02912-9 (PMC12539114; doi:10.1186/s40359-025-02912-9)
Supplement: Supplementary file 1 — Supplementary Material 1 [file 40359_2025_2912_MOESM1_ESM.pdf]

EMPOWER

Focus Group Interview Script
(ref. 101060918)

Study topic:

Assessing and fostering cognitive and emotion-related competencies in children with neurodevelopmental disabilities.

Interview's main objectives

To know the participants' perceptions about the cognitive abilities of children with neurodevelopmental disabilities.

To identify participants' perceptions about children with neurodevelopmental disabilities' emotion regulation.

To acquire information about tasks which may help develop the cognitive abilities of children with neurodevelopmental disabilities.

To acquire information about tasks which may help develop more adaptive emotion regulation strategies of children with neurodevelopmental disabilities.

To acquire information about technological resources which may help develop the cognitive abilities of children with neurodevelopmental disabilities.

To acquire information about technological resources which may help develop more adaptive emotion regulation strategies of children with neurodevelopmental disabilities.

Study variables

Cognitive factors	*Emotional factors*
Working Memory	Emotion Naming
Inhibitory Control	Emotion Intensity
Attention Shifting	Emotion Understanding
Cognitive Flexibility	Emotion Regulation Strategies
Delay of Gratification	

Approximate duration of the interview: 60 minutes

Block A - Objective: To legitimize the interview (5 min)

Inform the interviewees about the work to be carried out, the relevance of the study and its objectives.

Objective: To present the interviewer and the project.

Hi, my name is (interviewer's name) and I'm a psychology intern. I come from the Faculty of Psychology of the University of (interviewer's university). The interview I am doing with you is for a research project on the cognitive and emotional factors of children with neurodevelopmental disabilities. Therefore, I would like to talk to you about the project EMPOWER, funded by the Horizon Europe program (ref. 101060918).

Objective: To request the interviewees' collaboration.

Your collaboration is very important because it will help us to better understand the cognitive and emotional factors involved in children with neurodevelopmental disabilities' learning and the technological tools and tasks that can be created to help these children.

EMPOWER

Objective: To ensure the confidentiality of the interview and data processing.

Provide the following information regarding ethical procedures: Everything you say in this interview is confidential and the privacy of the participants in the processing of data is guaranteed. You can withdraw and stop the interview at any time. As support for the investigation, it is important to record your answers in writing and audio, so that it is possible to listen to them again and confirm some information. All records will be deleted after data processing, results will not be disclosed outside the scope of this investigation, and only global results will be disclosed, respecting the privacy of each participant.

Objective: To request permission to record the interview in audio format.

Question 1. Do you agree that I make an audio recording of the interview and make written notes?

If any of the respondents' state that they do not: it should be explained better that the audio recording and in writing is to facilitate our work since it is difficult to remember the opinions that are very important for our work. If the interviewee still does not authorize it, they are asked to just allow taking notes (e.g., single words/keywords/expressions).

Inform the participants of the following: We need to gather information regarding the cognitive abilities of children with neurodevelopmental disabilities, as well as tools to help these children improve these abilities. Therefore, we greatly appreciate your answers to the following questions, please.

Block B - Objective: To question about cognitive factors, as well as tools which may aid children with neurodevelopmental disabilities

Main questions	Secondary questions
**Question 1**: What are NDDs in your opinion?	**Comment to question 1:** After they answer, we should provide them our definition of NDD (based on DSM5)
**Question 2**: Children with NDDs, as you may know can have difficulties in i) the ability to memorize under distractors, ii) the ability to maintain and change attention, iii) the ability to wait for gratification, iv) behaving appropriate in specific contexts; v) have flexibility to switch from one task to another. Could you give us some examples of regular schools' tasks in which these abilities are needed?	**Comment to question 2**: if they don't provide answers for each specific abilities (i-v) ask them specifically for the ability that was not brought up.
**Question 3**. What type of computerized games could you suggest that assess the ability to memorize with the interference of other stimuli or the ability to organize	

EMPOWER

previously memorized information? (working memory)?	
**Question 4:** Please share with me some suggestions of computerized games to assess their ability to resist the impulse to immediately acquire rewards as opposed to obtaining them later after completing tasks successfully (delay of gratification)?	
**Question 5.** Could you please suggest a task that requires stopping an ongoing response when signaled to do so or when feedback suggests that the response is ineffective or maladaptive? (inhibitory control)	
**Question 6:** Which type of computerized tasks would you suggest to improve children with NDDs ability to adapt to different tasks more easily? (cognitive flexibility)	
**Question 7:** What type of computerized task can help children with NDDs to improve sustained attention, in terms of concentrating on a specific task for longer period of time? (sustained attention)	

EMPOWER

Inform the participants of the following: Considering there is sometimes a disconnect between self-reported emotion and expected physiological patterns in children with neurodevelopmental disabilities, we greatly appreciate your answers to the following questions, please.

Block C - Objective: To question about emotional factors, as well as tools which may aid children with neurodevelopmental disabilities.

Main questions	Secondary questions
**Question 1:** Considering the connection between cognition and emotion, could you please give us some examples of situations that illustrate the relation between the two?	

**Question 2:** Could you give us some examples of computerized tasks that could help children name and recognize their emotions?	
**Question 3:** Name some examples of computerized tasks that can assess and train the underlying difficulties of children NDDs in paying attention to and being aware of how they feel (emotional/awareness/ understanding)?	
**Question 4:** When children with NDDs feel unpleasant emotions for a long time, what can help them deal with the kind of emotions they are feeling? (Access to emotion regulation strategies)?	
**Question 5:** Name some examples of computerized tasks that can assess and train the use of adaptive emotion regulation strategies.	**Question 1:** What type of computerized tasks could help children with neurodevelopmental disabilities to select adaptative emotion regulation strategies in order to meet individual and social goals? (involvement in goals)<br><br>**Question 2**. What type of activity could help children with neurodevelopmental disabilities use compensatory emotion strategies when they are not able to control their emotions in order to do so? (emotion reappraisal)<br><br>**Question 3:** What type of computerized tasks can assess and model the acceptance and control of their own emotion?<br><br>**Question 4**: How can technology help children with NDDs think about or concentrating on other things while feeling unpleasant emotions?

EMPOWER

Question 6: What is your opinion on using computerized games to practice any of these abilities?	

Block D - Objective: To finish the interview (5 min)
Inform the interviewees about end of the interview.
**Objectives:** To understand if there is any other contribution that the interviewee wants to share; To reinforce ethical-deontological care that will be ensured; To mention the possibility of accessing the global results; To inform of the importance of the participants' collaboration in the project; To thank the participants for their availability.
**Question 1.** We are finishing our interview. Do you want to add something or is there any other relevant aspect you want to address?
**Question 2.** You may have access to general data from this first study. If you are interested, you can provide us with your email contact.
**Question 3.** We would like to thank you again for your availability and your contribution to the development of this project. We have reached the end of the interview.

EMPOWER
