# Supplementary material for: Neurodevelopmental disorders: assessing and training working memory
Source: BMC Psychol. 2025 Oct 21;13:1163. doi: 10.1186/s40359-025-02912-9 (PMC12539114; doi:10.1186/s40359-025-02912-9)
Supplement: Supplementary file 2 — Supplementary Material 2 [file 40359_2025_2912_MOESM2_ESM.pdf]

EMPOWER (ref. 101060918)

Focus Group with a Task - Interview Script

Study topic:
Assessing digital games on cognitive-related processes in children with neurodevelopmental disabilities.

Interview's main objectives
To know the participants' perceptions about integrating technological tools in the education process of children with neurodevelopmental disorders.
To acquire information about the adequacy of the games' characteristics.
To know the participants' perceptions about the adequacy of each game (working memory, sustained attention and inhibition) concerning cognitive competencies in children with neurodevelopmental disabilities.
To acquire information about the advantages and disadvantages in regard to the use of the technological games in training executive functions.
To itendity possible problems when using the games with children with neurodevelopmental disorders.
To acquire feedback about the usefulness and usability of the games.
To acquire feedback in regard to most suitable end-users and how the feedback is provided to end-users.

Study variables
Game Characteristics
Game Objectives
Main foreseeable problems
Adequacy of the games to children's characteristics
Usefulness of the games
Advantages and Disadvantages
Gameplay Enjoyment
Usability in classrooms

Approximate duration of the interview: 60 minutes

Block A - Objective: To legitimize the interview (5 min)
Inform the interviewees about the work to be carried out, the relevance of the study and its objectives.
**Objective: To present the interviewer and the project.**
Hi, my name is (interviewer's name) and I'm a psychology intern. I come from the Faculty of Psychology of the University of (interviewer's university). The interview I am doing with you is for a research project on the cognitive and emotional factors of children with neurodevelopmental disabilities. Therefore, I would like to talk to you about the project EMPOWER, funded by the Horizon Europe program (ref. 101060918).
**Objective: To request the interviewees' collaboration.**
Your collaboration is very important because it will help us to better understand the adequacy of the technological game that we have been developed on cognitive and emotion-related competencies in children with neurodevelopmental disabilities.
**Objective: To ensure the confidentiality of the interview and data processing.**

Provide the following information regarding ethical procedures: Everything you say in this interview is confidential and the privacy of the participants in the processing of data is guaranteed. You can withdraw and stop the interview at any time. As support for the investigation, it is important to record your answers in writing and audio, so that it is possible to listen to them again and confirm some information. All records will be deleted after data processing, results will not be disclosed outside the scope of this investigation, and only global results will be disclosed, respecting the privacy of each participant.
**Objective: To request permission to record the interview in audio format.**
**Question 1.** Do you agree that I make an audio recording of the interview and make written notes?
If any of the respondents state that they do not: it should be explained better that the audio recording and in writing is to facilitate our work since it is difficult to remember the opinions that are very important for our work. If the interviewee still does not authorize it, they are asked to just allow taking notes (e.g., single words/keywords/expressions).

Inform the participants of the following: We need to gather information regarding the appropriateness of each game concerning the difficulty level, adequacy of the response time, general characteristics of the game, and specific characteristics such as the appropriateness of the colors used in the game…

Block B - Objective: To introduce the theme about technology	
**Question 2:**<br>What do you think about integrating technological tools in the education process of children with neurodevelopmental disorders?	**Question 2a:**<br>Have you ever used technology in your regular classes? Please give examples.

Block C - Objective: To get participants to play and reflect about the games
**Focus Group Interview Task**
**Resources –** Reflection Sheet, Tablet with Working Memory Game, Inhibition Game an Sustained Attention Game.
**Procedures –**<br>○ Give one of the group members the Reflection Sheet where they can take notes/ register colleagues’ ideias, etc.<br>○ Have one of the group members play the first game. They can then swap and each member plays a different game / level, or the same member can play all the games and the remaining group members observe.
**Information for the participant:**<br>I would like you to try three games that we are developing and let us know what you think as you are playing the games. You can make verbalizations and you go along and take notes as a group on the sheet I have given you. We really appreciate your reflections.

EMPOWER

Block C - Objective: To gather participants' reflections about the games	
**Question 3:**<br>Please share with me your thoughts about the game and the notes you wrote down while playing the game.	
**Question 4:**<br>Do you foresee any problems when assessing and training children's with these games? If so, explain which.	
**Question 5:**<br>Considering the children who you work with, which are the characteristics (what type of children, in terms of age, diagnosis, cognitive abilities) that we should consider when recruiting children in our studies and who you think will benefit most from them?	
**Question 6:**<br>How useful do you consider the games, in terms of assessing and training executive functions? What other types of cognitive processes and behavior do you believe that this training could help?	**Question 6a**:<br>Do you think the games can predict these children's academic performance, and/or social and emotional competencies?
**Question 7:**<br>How could you use the information extracted from the games in your everyday work?	
**Question 8:**<br>How should feedback be given to the children through the games?	

Block D - Objective: To finish the interview (5 min)
Inform the interviewees about end of the interview.
**Question 9.** We are finishing our interview. Do you want to add something or is there any other relevant aspect you want to address?
**Question 10.** You may have access to general data from this first study. If you are interested, you can provide us with your email contact.
**Question 11.** We would like to thank you again for your availability and your contribution to the development of this project. We have reached the end of the interview.

EMPOWER
