# Supplementary material for: Neurodevelopmental disorders: assessing and training working memory
Source: BMC Psychol. 2025 Oct 21;13:1163. doi: 10.1186/s40359-025-02912-9 (PMC12539114; doi:10.1186/s40359-025-02912-9)
Supplement: Supplementary file 3 — Supplementary Material 3 [file 40359_2025_2912_MOESM3_ESM.pdf]

REFLECTION SHEET FOR FOCUS GROUP INTERVIEW WITH A TASK

Register the group members' reflections about each game as one member is playing.

WORKING MEMORY GAME (10 minutes)

General comments: advantages, disadvantages, usefulness, enjoyment, and suggestions.

Circle your response for each item.

- **To measure working memory in children with neurodevelopmental disorders:**

- The game reaches its objective: *disagree / agree more or less / agree*
- The game has an appropriate range of difficulty: *disagree / agree more or less / agree*
- The response time is adequate: *disagree / agree more or less / agree*
- The colors are appropriate: *disagree / agree more or less / agree*
- *gree*

EMPOWER
